# Randall Packard: learning to learn from global health history

**DOI:** 10.2471/BLT.18.030418

**Published:** 2018-04-01

## Abstract

The World Health Organization marks its 70 years this month on 7 April. Randall Packard tells Fiona Fleck why today’s global health leaders can learn from the past.

Q: How did you first become interested in global health and its history?

A: I was in the Peace Corps in Uganda in the late 1960s and 1970s in the trachoma eradication programme. Like many other disease control programmes that were initiated in that period and later, it was led basically by a nongovernmental organization (NGO) that had little connection with the ministry of health. There were no provisions to train local people to do our job, so that they could replace us when our tour was over. Thus, the project could not be sustained after we left the country. When I returned from Uganda I completed a PhD in African history and became interested in the history of disease in Africa. I worked on the history of tuberculosis and malaria in southern Africa and this led me to an interest in how international health organizations designed and implemented health interventions.

Q: Can you briefly describe your work on the history of global health?

A: My initial interest in global health focused on malaria. I wrote about the WHO-led malaria eradication campaign and more recent efforts to eliminate malaria through Roll Back Malaria in my book *The making of a tropical disease: a short history of malaria*. My most recent book *A history of global health, interventions into the lives of other peoples *examines the history of international health from the early 20th century to the present.

Q: Why is the study of the history of global health important?

A: At a general level, history provides a corrective to the kind of historical narratives that people within global health produce. When I was at Emory, I had a joint teaching appointment in history and international health, and spent a lot of time working with people from the United States Centers for Disease Control and Prevention (CDC). At Johns Hopkins I have an appointment in the History of Medicine department and in the Bloomberg School of Public Health. My work with international health professionals made me aware that they often produce histories that explain why various policies were chosen, and why they succeeded or failed. These histories tend to validate their work. They tend to ignore the messiness of history and the role that political and economic factors play in shaping policy decisions and determining whether programmes succeed or fail. One of my main goals in teaching the history of global health has been to encourage those entering the field to think critically about these narratives and to better understand the wider political and economic contexts in which global health programmes occur.

“Public health professionals often produce histories that … tend to validate their work.”

Q: For example?

A: Before the Global Malaria Eradication Programme (GMEP) was launched in 1955, several malaria pre-eradication projects were conducted in West Africa in the early 1950s. The projects sought to use dichlorodiphenyltrichloroethane (DDT) to eliminate malaria, lasted a few years, reduced malaria transmission, but failed to eliminate the disease. The programmes were shut down and the spraying stopped. When this happened, malaria rebounded because people living in highly endemic areas lost their acquired immunity to the disease. When exposed again to intense malaria transmission, they often suffered severe, even deadly bouts of the disease. This experience should have been a lesson for those planning the 1955-69 GMEP. If you try to eradicate malaria and fail, the costs in human lives can be high. However, this historical lesson was not heeded and when the GMEP funding ran out and countries cutback on control efforts, malaria resurged dramatically, particularly in India, Sri Lanka and the highlands of Madagascar.

Q: Are there other examples where global health policy would have benefited from learning from history?

A: The smallpox eradication programme – unlike malaria – was a tremendous success that inspired a series of eradication and elimination efforts, some of which continue today. However, smallpox is unique. The epidemiological characteristics of the disease and the effectiveness of the vaccine made it possible to eradicate smallpox without investing in health services or improving social and economic conditions. Smallpox may be the only disease that is possible to eradicate without addressing socioeconomic and other contextual factors. More broadly, the success of smallpox eradication has empowered an increased reliance on biomedical technologies to address global health problems. Insecticide-treated bednets, vaccines and antiretroviral drugs are examples of the increased medicalization of global health. I am not saying these interventions have not had a positive impact. They have. Rather I am suggesting that this focus on technology has occurred at the expense of investments in basic health services and in addressing the social determinants of ill health. A better historical understanding of why smallpox eradication was successful might have led policy makers to consider this when searching for solutions to global health problems.

“This focus on technology has occurred at the expense of investments in basic health services.”

Q: We tend to think of historians as reading and doing archival research, how useful was your work in African countries for the study of global health?

A: Living in the Democratic Republic of the Congo, South Africa, Swaziland and Uganda was essential. Often global health policies are made from the top down, with limited input from people working on the ground. Working in countries in Africa gave me a much better sense of the impact global programmes have and how they affect people’s lives. Not only are global health programmes top-down, but often there is no accountability. People in these countries are the targets of the programmes with little role in shaping them. When the programmes fail, the people affected often feel abandoned. Many global health programmes have short shelf-lives. Suddenly, funds are switched to another programme and countries must scramble to accommodate them.

Q: For example?

A: When I was in Swaziland there was a big push to deal with tuberculosis, but within a couple of years international organizations decided that they needed to invest in waterborne disease projects following an outbreak of cholera. When this happened, support for tuberculosis programmes dried up. When working on the ground, you realise that countries have limited resources and they are very dependent, so the donors call the shots for the most part. Everyone involved in global health decision-making should be required to work in the countries and see how things look from the ground level.

Q: WHO's initial drive to promote primary health care as a route to health for all by 2000 did not deliver on its pledge, why?

A: The primary health care movement did not achieve the goals set out in 1978 at the Alma Ata conference for several reasons. Firstly, the vision of what could be achieved was idealistic, as it assumed the existence of democratically run village communities in which popular participation, a central element in primary health care, could be easily achieved. In reality most communities were hierarchical and patriarchal. Secondly, there was no blueprint on how to implement primary health care and how to move forward or deal with the opposition from the medical establishment. Shifting resources from tertiary to primary care was never easy. Thirdly, a few years after the Alma Ata declaration, there was a global economic recession. This limited the resources available to fund the building of primary health programmes. It also coincided with an emerging neoliberal economic agenda and the Washington consensus, driven by the World Bank, in which large-scale public funding of global health programmes was reduced and there was a shift to self-funding and “selective” primary health care, a new approach that valued efficiency and cost-effectiveness over coverage and quality of basic health services. Structural adjustment policies were introduced that tended to undermine rather than strengthen health systems. It was a powerful ground shift that made health for all unlikely and difficult to implement.

Q: What are the lessons from the history of the drive for health for all?

A: As mentioned, the approaches that emerged from the smallpox eradication programme of the 1960s and 1970s drove global health towards a more technocratic approach, narrowly focused on specific diseases and public health fields. The main lesson for the World Health Organization (WHO) is that after the 1976 health for all declaration, there were limits to what could be achieved, given this political and economic environment. There is a real need for investment in basic health services. The failure to do this has been a central theme in the history of international and global health from the early 20th century to the present.

Q: What are the origins of global public health? How is global public distinct from international public health?

A: The concept of global health emerged in the late 1980s. In my book on the history of global health and its origins, I argue that it’s both old and new. Some trends continue: global health decision-making occurs in places that are distant from where projects are implemented; global heath accountability goes upward and not downward; there is a lack of attention to the economic and social determinants of health; investments in technocratic solutions are favoured over investments in health systems. On the other hand, the massive funding increase for global health and shift in the players in this field are new.

Q: How are new players changing global health?

A: In addition to WHO, CDC and the United Nations Children’s Fund, you have the World Bank, the Gates Foundation, the Global Fund to Fight AIDS, Tuberculosis and Malaria and public private partnerships like Roll Back Malaria and GAVI, the vaccine alliance, which have different agendas and approaches, and are less accountable than WHO, which has country representation. In some ways, global health decision-making, which has always been external to the countries where decisions have an impact, has moved even further away from those countries. Moreover, the new players have introduced new demands for efficiency and performance-based lending. There is a greater emphasis on evidence-based programmes supported by scientific research. All of this favours technological fixes over broad-based investments in basic health-care services, clean water and sanitation – interventions which are harder to quantify. There is a lot that is new about global health. 

